# Snoezelen or Controlled Multisensory Stimulation. Treatment Aspects from Israel

**DOI:** 10.1100/tsw.2004.30

**Published:** 2004-05-11

**Authors:** Joav Merrick, Carmit Cahana, Meir Lotan, Isack Kandel, Eli Carmeli

**Affiliations:** National Institute of Child Health and Human Development, Office of the Medical Director, Division for Mental Retardation, Ministry of Social Affairs, Jerusalem, Israel; ^2^ Zvi Quittman Residential Center, The Millie Shime Campus, Elwyn Jerusalem, Israel; ^3^ Faculty of Social Science, Department of Behavioral Sciences, Academic College of Judea and Samaria, Ariel, Israel; ^4^ Department of Physical Therapy, Sackler Faculty of Medicine, Stanley Steyer School of Health Professions, Tel Aviv University, Israel

**Keywords:** mental retardation, developmental disability, intellectual disability, human development, public health, Snoezelen, Israel

## Abstract

In Israel today, with a total population of over 6 million persons, the Division for Mental Retardation (DMR) provides services to 23,000 persons with intellectual disability (ID). Of the 23,000, residential services are provided to more than 6,000 in close to 60 residential centers, another 2,000 are provided residential care in hostels or group homes in the community in about 50 locations, while the rest are served with day-care kindergarten, day-treatment centers, sheltered workshops, or integrated care in the community. The first Snoezelen room (controlled multisensory stimulation) in the DMR was established at the Bnei Zion residential care center in 1995. The Snoezelen method is now used in Israel in more than 30 residential care centers and 3 community settings. Since the year 2000, a physiotherapist has been employed in order to supervise the treatment and development of the method nationally. Professional staff meetings take place every 4 months. A certification course has been established on a national basis for individuals from different professions (occupational therapists, physiotherapists, teachers, music therapists, nurses, speech therapists, or caregivers). Snoezelen has proved to be an important instrument and a powerful therapeutic tool among the various treatment modules employed in Israel for persons with ID. This paper presents the concept illustrated with two case stories.

## INTRODUCTION

The first residential home for individuals with intellectual disability (ID) in Israel was established in 1931 as a boarding school for a small number of children. A larger residential care center was later established in Herzliya in 1945[1]. From the establishment of the modern State of Israel in 1948 until 1961, the Ministry of Welfare was responsible for the care of persons with ID through services provided by the Child, Adolescent and Youth Protection Services. In 1962, the Services for the Mentally Retarded was established and in 1977 upgraded to a Division for Mental Retardation (DMR) under the newly established joint Ministry of Labour and Social Affairs (in 2003 again divided back into two ministries) with the responsibility for the assessment, treatment, and habilitation of persons with ID.

Israel today has a total population of 6,037,000 persons and the DMR is responsible for the care of 23,000 persons (all ages). Of these 23,000 persons, residential care is provided to about 6,000 individuals in close to 60 centers throughout the country. In addition, 2,000 individuals benefit from other residential care, such as hostels and protected apartments in 50 locations in the community. The rest (15,000) are living at home, but are served with day-care kindergarten, day-treatment centers, sheltered workshops, or integrated care in the community. The age distribution in the year 2002 of 6,352 persons with ID in residential care centers is presented in Table 1 and the level of ID is presented in Table 2[2]. There were 9 governmental institutions, 36 private institutions, and 12 public institutions with a mean of 111.44 (range 16–398) persons in each center. The total expenditure for all residential care centers is provided by the government, which also manages the governmental institutions, whereas the private and public institutions have their own administration, but are provided with budget, clients, and supervision by the government. The annual expenditure of the DMR is over US$200 million[3].

**TABLE 1**
**The Population of Persons with ID in Residential Care Centers in Israel 2002[2]**

| Age (in years) | Males | Females | Total | Percent |
|---|---|---|---|---|
| 0–9 | 100 | 69 | 169 | 2.66 |
| 10–19 | 475 | 372 | 847 | 13.33 |
| 20–39 | 1,589 | 1,078 | 2,667 | 41.99 |
| 40–49 | 721 | 691 | 1,412 | 22.23 |
| 50–59 | 463 | 436 | 899 | 14.15 |
| >60 | 166 | 192 | 358 | 5.64 |
| Total | 3,514 | 2,838 | 6,352 | 100.00 |
| % | 55.3 | 44.7 | 100.00 | |

## PHYSIOTHERAPY SERVICE FOR PERSONS WITH INTELLECTUAL DISABILITY IN RESIDENTIAL CARE

Before 1991, the physiotherapy service of the DMR was very irregular and many residential care centers were without a physiotherapist. In 1994, the Medical Director of the DMR appointed a Chief Physiotherapist responsible for general issues and manpower and another physiotherapist as a coordinator for the provision of wheel chairs, seating and positioning.

Today, physical therapy services are provided by 80 full- or part-time physiotherapists in 57 residential care centers with the equivalent of 45 full-time physiotherapist positions. The average annual time spent per person in 2002 was 12.5 h and the average annual treatment sessions per person was 11.7[2]. It should be noted that not everyone in the residential care centers receive physiotherapy, so in

**TABLE 2**
**The Level of ID in the Population of Persons with ID in Residential Care in Israel 2002**

| Age (in years) | Mild | Moderate | Severe | Profound | Other | Total | Percent |
|---|---|---|---|---|---|---|---|
| 0–9 | 3 | 11 | 98 | 57 | 0 | 169 | 2.66 |
| 10–19 | 42 | 302 | 354 | 146 | 3 | 847 | 13.33 |
| 20–39 | 169 | 1,009 | 1,017 | 466 | 6 | 2,667 | 41.99 |
| 40–49 | 132 | 680 | 456 | 136 | 3 | 1,407 | 22.23 |
| 50–59 | 95 | 458 | 256 | 85 | 4 | 898 | 14.15 |
| >60 | 28 | 200 | 92 | 39 | 5 | 364 | 5.64 |
| Total | 469 | 2,660 | 2,273 | 929 | 21 | 6,352 | 100.00 |
| Percent | 7.38 | 41.88 | 35.78 | 14.63 | 0.33 | 100.00 | |

Mild ID: IQ 55–70, moderate: IQ 35–54, severe: IQ 20–34, and profound: IQ <20. Other are 21 persons who either historically were placed in institutions for other reasons and preferred to stay on because they regarded the institution as their home or persons under observation.

reality, the persons in need of physiotherapy received more. To compare this information to the general population, every person in need of physiotherapy is entitled to 12 treatments per year provided by their HMO or Sick Fund (each treatment is of 30-min duration).

In each residential care center where there is the need for a physical therapy intervention, a specially equipped room has been built for this purpose. Also, in several large residential centers, physiotherapy rehabilitation centers have been established. The physiotherapy services are state of the art, updated, and geared toward primary, secondary, and tertiary prevention.

## SNOEZELEN /MULTISENSORY STIMULATION

The concept of "Snoezelen" or controlled multisensory stimulation was introduced by staff working at two Dutch residential centers for persons with ID in the 1970s[4,5]. The word "Snoezelen" is a combination of two Dutch words: *snuffelen* or the sniffing of a dog and *doezelen* meaning to slumber, relax, or doze[4,5]. The term Snoezelen refers to a specially equipped room(s), where the management (i.e., nature, quantity, arrangement, and intensity of stimulation**)** is controlled[5] with an environment designed to stimulate the senses by means of light, sound, touch, smell, and taste[6]. The founders of the concept[4] used the method as a recreational or leisure activity without therapeutic elements or supervision and initially resisted any notion of research in the fear that it would become more therapeutic, objective, and product oriented[7]. This notion is gradually changing and a recent review[7] identified 21 research studies published on this subject, where 14 studies involved persons with ID and 7 studies involved people diagnosed with dementia. Of these 21 studies, 14 reported positive effects within the session in the Snoezelen room, 4 showed immediate positive postsession effects, 2 long-term effects and one inconclusive. The effects measured relied on qualitative and unstructured data, such as postsession ratings, diary cards filled in afterwards, and staff interview[7]. One study from the United Kingdom[8] followed 27 adults with severe and profound ID over a 16-week period (two weekly Snoezelen sessions) with a control group and they were unable to observe any effect beyond that ascribed to the social interaction between the participant and the therapist. The level of evidence of the positive effects is therefore preliminary and not based on solid research evidence for which there is an urgent need.

The concept of a multisensory environment was based on the assumption that the world is a mixture of light, sound, small, taste, and tactile sensations that we feel through our sensory organs[4]. With cognitive impairment (either dementia or ID), behavioral disturbances will follow (like agitation or aggression) and the original idea was to use the multisensory environment to function as leisure and relaxation to calm the patient.

## SNOEZELEN /MULTISENSORY STIMULATION IN ISRAEL

The Snoezelen method was brought to Israel in a day-care treatment center for children with ID (Beit Issie Sharipa, Raanana) in 1993 by Michele Shapiro[5]. The first Snoezelen room in the DMR was established at Bnei Zion (Rosh Haayin) residential care center in 1995 and today the Snoezelen method is used in more than 30 residential care centers and 3 community settings.

Since the year 2000, a physiotherapist (national coordinator) has been employed by the Medical Director of the DMR to supervise the treatment and the development of the method nationally. Professional meetings among the staff involved take place every 4 months on-site in one of the residential care centers with a Snoezelen room, and an accreditation course (40 credit hours) has been established on a national basis for the different professionals involved (occupational therapists, physiotherapists, teachers, musical therapists, nurses, speech therapists, or caregivers). The first handbook[5] for practitioners was published in English in 2002.

In addition to the Ministry of Social Affairs, the Ministry of Education introduced Snoezelen as a treatment concept for students enrolled in special education programs in several locations in the year 2000, and recently several nursing homes for the elderly and rehabilitation institutions for the disabled have introduced Snoezelen as well. The major medical center for children in Israel, “Schneider Children’s Medical Center” in Petach Tiqva, has also established Snoezelen in their surgical department.

## SNOEZELEN AND THE DIVISION FOR MENTAL RETARDATION

Today the DMR of the Ministry of Social Affairs operates 31 Snoezelen rooms around the country. In 20 Snoezelen rooms, allied health professionals operate the facility and in 25 residential care centers, there are several therapists of different professions in the room together with the client(s). This approach gives a multidisciplinary dimension to the treatment.

Today therapists are permitted to use the Snoezelen method only after passing the national accreditation program and participation in the professional meetings. During these meetings, case studies, personal experiences, and research issues are discussed. These meetings take place in different locations in order to make site visits and learn from that experience and various approaches to the Snoezelen treatment.

## CONCEPT

The concept of Snoezelen was introduced in residential care centers in Israel for persons with ID in order to add to the variety of treatment possibilities for this population. It was seen as another instrument for communication between therapist/caretaker and client. As seen in other countries also[7], this treatment module has become popular due to the limited number of alternative resources available for care staff to work with severely disabled people and the approach can be seen as pleasurable, friendly, and humane[7].

The concept has also made it possible for multidisciplinary interaction between care staff and professionals. In one residential care center, the occupational therapist started working with the social worker within the Snoezelen room, with the whole family of the institutionalized child. In Israel, in contrast to Holland where the concept is recreational, the focus is mainly therapeutic and therefore assessment of the client before, during, and after treatment in the Snoezelen room has become mandatory. The environment in the Snoezelen room is created in accordance with the need and desires of the clients, but the therapist is there to interact with the client so that the time spent in the room is a more structured and active.

Each residential care center in Israel has the flexibility to elect the treatment model they prefer and decide on the professional personal that will work in the room. The national coordinator is available for advice and supervision. They can also decide which clients are suitable for the treatment in the Snoezelen room. The DMR has provided guidelines for the treatment and safety while working in the room. Each residential care center has conducted proper assessments before, during, and after treatment. At the moment, work is being done to create national standard guidelines for an assessment instrument and follow-up scale.

Snoezelen treatments are usually limited to 30 min and although most of the treatments today are individualized, some centers have created group work and even family therapy in the room.

## CASE STUDIES

Each client in the Snoezelen treatment at the Zvi Quittman Residential Center is treated twice a week with 30-min sessions. They are treated according to the work schedule of the care staff and in both case studies the sessions took place in the morning. Each caregiver has passed the national accreditation course and is supervised by a physical therapist. At first, supervision was given regarding each client and for general manual therapy guidance. This form of supervision was given until the caregiver reached a point where constant supervision was no longer needed. From this point on, supervision is given whenever a caregiver asks for supervision, or whenever a therapeutic plan is introduced to new clients.

Pretreatment evaluation was done a month before intervention initiation to the multisensory environment. Each client is evaluated with regard to the target behaviors that will be a measure for treatment success (for example: self injurious behavior, food snatching, cloth tearing, shouting or hitting others). From this point on, the staff is requested to fill out a standard weekly form that measures the occurrence of the target behavior. With the progression of the intervention plan, the occurrence of the target behavior is constantly evaluated by the physical therapist. Results and the decision to continue treatment are then re-evaluated by the therapeutic team every 3 months according to the change in target behavior.

Every client starting intervention in the multisensory environment is first introduced to the room with his/her caregiver (who will later perform the on-going therapy sessions) and with the physical therapist. The room is introduced to the client, where the preferences and dislikes are observed for use later to build a treatment plan. This process usually takes 1 h to complete, but could take up to five 1-h sessions according to compliance of the client and the experience of the therapist.

### Case Study 1

Female, 32 years old, with severe ID of unknown origin, living for the past 20 years in the residential care center. She has shown challenging behaviors manifested in refusal to participate in group activities and severe feeding problems.

The physiotherapist and care staff assessed the woman and created a treatment plan in order to change the behavior. The target behaviors (the amount of refusal occasions) were monitored starting 1 month prior to treatment initiation (continuously being measured).

The treatment in the Snoezelen included back massage and relaxation techniques. Positive changes in food consumption and participation in activities were identified after only 1 week of treatment. After 2 months of treatment, a weight gain of 3 kg was observed (see Fig. 1) and supplementary nutrition therefore discontinued (positive cost-benefit).

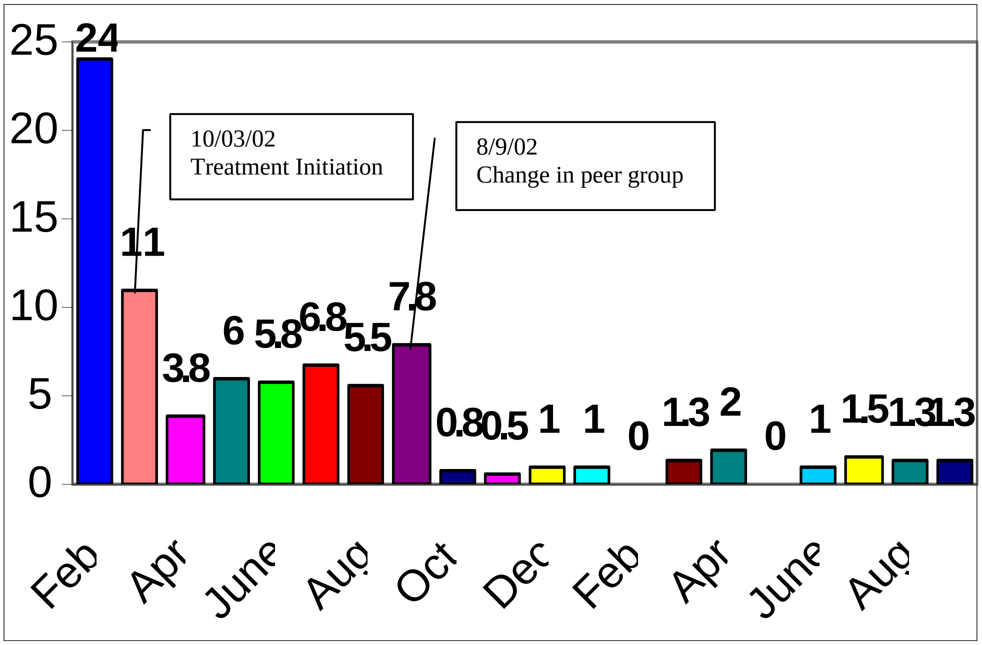

**FIGURE 1.** Challenging behavior before and during Snoezelen therapeutic sessions.

## Case Study 2

Female at the residential center, 53 years old, was showing challenging behaviors manifested in refusal to participate in hygiene activities and unexplainable episodes of constant shouting periods that lasted for many hours each day. These episodes were disturbing for her peer group and care staff. Shouting episodes were monitored a month prior to treatment initiation (continuously being measured). A treatment plan in the Snoezelen was built and the therapeutic sessions executed. Slow reduction in shouting episodes was observed (as shown in Fig. 2), while other challenging behaviors (hygiene problems) discontinued after a few therapy sessions. The positive changes are still lasting after a period of more than 2 years.

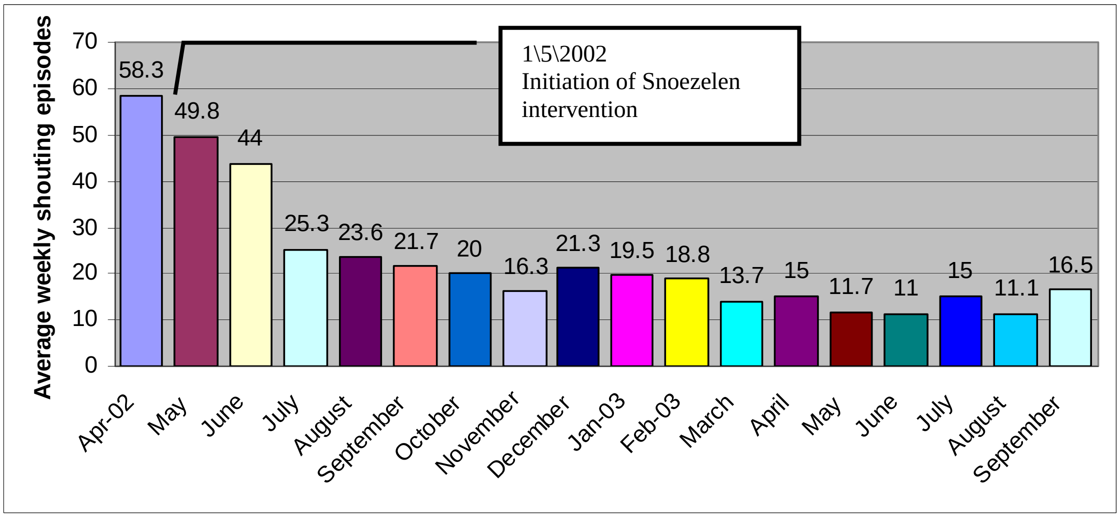

**FIGURE 2**. Shouting episodes before and after initiation of Snoezelen intervention.

## CONCLUSIONS

Snoezelen or controlled multisensory stimulation was brought to Israel in 1993 and the first room established in a residential care center for adults with ID in 1995. The concept in Israel has been with a focus on treatment and we have seen many positive rehabilitative aspects over the past years for the benefit of the clients served.

## BIOSKETCHES

**Joav Merrick, MD, DMSc,** is Professor of Child Health and Human Development affiliated with the Zusman Child Development Center, Division of Pediatrics and Community Health at the Ben Gurion University, Beer-Sheva, Israel; the Medical Director of the Division for Mental Retardation, Ministry of Social Affairs, Jerusalem; and the Founder and Director of the National Institute of Child Health and Human Development. He has numerous publications in the field of child and human development, rehabilitation, intellectual disability, disability, health, welfare, abuse, advocacy, quality of life, and prevention. Dr. Merrick received the Peter Sabroe Child Award for outstanding work on behalf of Danish Children in 1985 and the International LEGO-Prize ("The Children's Nobel Prize") for an extraordinary contribution towards improvement in child welfare and well being in 1987. E-mail: jmerrick@internet-zahav.net. Website: www.nichd-israel.com

**Carmit Cahana**, **MScPT**, is the chief physiotherapist at the Office of the Medical Director, Division for Mental Retardation, Ministry of Social Affairs, Jerusalem, Israel. Her research interests include the role of the physiotherapist and persons with intellectual disability, assistive technology, Snoezelen or sensory multistimulation, and service utilization. E-mail: carmit_c@netvision.net.il

**Meir Lotan, MScPT,** is a physiotherapist working at the Zvi Quittman Residential Center, The Millie Shime Campus, Elwyn Jerusalem with special interest in physiotherapy aspects on intellectual disability, Snoezelen, and physical activity for children and adults with intellectual disability. He lectures on assistive technology at the Department of Physical Therapy, Haifa University and Ben Gurion University. E-mail: ml_pt_rs@netvision.net.il

**Isack Kandel**, **MA, PhD,** is Senior Lecturer at the Faculty of Social Sciences, Department of Behavioral Sciences, the Academic College of Judea and Samaria, Ariel. During the period 1985–93, he served as the Director of the Division for Mental Retardation, Ministry of Social Affairs, Jerusalem, Israel. E-mail: Kandeli@aquanet.co.il

**Eli Carmeli, BPT, PhD**, is currently a Senior Lecturer of Gerontology and Anatomy and the Chairman of the Department of Physical Therapy Department, Stanley Steyer School of Health Professions, Sackler Faculty of Medicine, Tel Aviv University. He is also the Editor-in-Chief of the *Israeli Journal of the Physiotherapy Society*. Eli received his BPT degree from Sackler Faculty of Medicine, Tel Aviv University and his PhD degree from the Rappaport Faculty of Medicine at the Technion in Haifa. Postdoctoral research was conducted at the University of Florida in Gainesville. His research interests are investigating the aging process both on the cellular and clinical level. E-mail: elie@post.tau.ac.il Website: http://www2.tau.ac.il/Person/medicine/HealthSchool/researcher.asp?id=agfhfiffl
